# Bending stiffness of *Toxoplasma gondii* actin filaments

**DOI:** 10.1016/j.jbc.2024.108101

**Published:** 2024-12-18

**Authors:** Wenxiang Cao, Thomas E. Sladewski, Aoife T. Heaslip, Enrique M. De La Cruz

**Affiliations:** 1 Department of Molecular Biophysics and Biochemistry, Yale University, New Haven, Connecticut, USA; 2 Department of Molecular and Cell Biology, University of Connecticut, Storrs, Connecticut, USA

**Keywords:** actin, filament, cytoskeleton, cell motility, microtubule

## Abstract

Actin is essential for the survival and pathogenicity of the Apicomplexan parasite *Toxoplasma gondii*, where it plays essential functions in cargo transport, invasion, egress, and organelle inheritance. Recent work has shown that, unlike vertebrate skeletal muscle actin, purified *T*. *gondii* actin filaments (TgAct1) can undergo rapid treadmilling, due to large differences in the barbed- and pointed-end critical concentrations, rapid subunit dissociation from filament ends, and a rapid nucleotide exchange rate constant from free monomers. Previous structural analysis suggested that the unique assembly properties of TgAct1 filaments may be a functional consequence of reduced contacts between the DNAse-1–binding loop (D-loop) of a filament subunit and its adjacent, long-axis subunit neighbor. Because the D-loop makes stabilizing interactions between neighboring subunits, it has been implicated in regulating the mechanical properties of actin filaments. In this study, we measured the bending persistence length (*L*_B_) of TgAct1 filaments and the filament length distribution. We found that despite compromised intersubunit D-loop contacts, TgAct1 filaments have similar bending stiffness and thermodynamic stability as vertebrate actin filaments. Analysis of published cryo-EM image density maps indicates that TgAct1 filaments retain a stabilizing intersubunit salt bridge between E168 and K62 and reveals visible density between Y167 and S61 of adjacent filament subunits, consistent with a conserved cation binding site proximal to the D-loop, as initially identified in vertebrate skeletal muscle actin filaments. These results favor a mechanism in which weak D-loop interactions compromise TgAct1 subunit incorporation at filament ends, while minimally affecting overall subunit interactions within filaments.

*Toxoplasma gondii* (*T*. *gondii*) is a single-celled parasite, capable of infecting a broad range of warm-blooded hosts. Chronic infection in humans causes Toxoplasmosis, a life-threatening disease in immunocompromised individuals or when infection occurs *in utero* (1, 2). *T*. *gondii* is a member of the larger phylum apicomplexan which includes other medically relevant parasites including *Plasmodium* spp. and *Cryptosporidium* spp., the causative agents of malaria and the diarrheal disease, cryptosporidiosis.

During a chronic infection, *T*. *gondii* replicates by repeated completion of its lytic cycle which involves host cell invasion, replication, and egress. Completion of the lytic cycle requires the expression of a divergent actin gene (TgAct1). TgAct1 is the sole actin isoform in *T*. *gondii* and shares only 83% identity with mammalian alpha (α), beta (β), and gamma (γ) isoforms (3). The function of TgAct1 is best studied in *T*. *gondii* for its role in a unique form of actomyosin-based cell motility known as gliding motility (4, 5, 6). However, more recently, the development of actin reporters that bind TgAct1 filaments has uncovered several other roles for actin in the parasite, including organelle inheritance (7), structural maintenance of the Golgi apparatus and endoplasmic reticulum tubules (8), cargo trafficking (9, 10), and the extrusion of a tubulin-based organelle at the apical end of the cell called the conoid (11, 12). Given the fundamental and diverse roles TgAct1 plays in parasite physiology, elucidating the biochemical, structural, and mechanical properties of TgAct1 filaments is needed to understand how TgAct1 senses and transmits the forces that drive these processes.

It was recently discovered that purified TgAct1 assembles into filaments with strikingly different properties compared to skeletal muscle actin (13). Actin monomers assemble into filaments when the total actin concentration exceeds the critical concentration ($a_{c}$) for assembly, which for ATP- and ADP-actin is defined as the free monomer concentration in reversible equilibrium with filament barbed and pointed ends at steady state. For ADP-actin, $a_{c}$ is also the dissociation equilibrium constant (*i*.*e*.*,* affinity *K*_d_ = *k*_−_/*k*_+_) for the monomer incorporation to filament ends, which is related to the standard free energy change (Δ*G*^o′^) for subunit incorporation by Δ*G*^o′^ = *RT*ln(*K*_d_). Direct visualization of *in vitro* TgAct1 filament assembly in the presence of ATP revealed a ∼70- to 150-fold higher observed critical concentration for polymerization at filament barbed and pointed ends compared to skeletal muscle actin (13, 14, 15). The TgAct1 barbed end critical concentration (*k*_−_^barbed^/*k*_+_ ^barbed^) has a value of ∼7 μM *versus* ∼0.1 μM for vertebrate skeletal muscle actin barbed ends, and a pointed end critical concentration (*k*_−_^pointed^/*k*_+_ ^pointed^) of ∼75–90 μM *versus* ∼0.6 μM for skeletal muscle actin filaments (13, 14, 15)). The large differences in the barbed- and pointed-end critical concentrations for polymerization, rapid subunit dissociation from filament pointed ends, and a rapid nucleotide exchange rate constant from free monomers allow TgAct1 filaments to undergo rapid treadmilling until solution ATP is depleted (13).

Cryo-EM structures of native *TgAct1* filaments with bound ADP show reduced contacts between the DNase-I–binding loop (D-loop) of a filament subunit and its longitudinal neighbor, potentially contributing to the higher critical concentration of TgAct1 filaments (13). These longitudinal subunit interactions have been implicated in regulating the assembly and mechanical properties of other actin filament types (16, 17, 18, 19, 20, 21). Here, we determine whether the reduced D-loop–mediated intersubunit contacts in the TgAct1 filament and the higher critical concentration influence the filament length distribution and bending stiffness.

## Results

### TgAct1 actin filaments have the same bending stiffness as skeletal muscle actin filaments

The flexural rigidity (*κ*), or bending stiffness (22), of slender rods such as actin filaments, defines their resistance to bending by thermal or applied forces (23). The flexural rigidity (*κ*) is related to the apparent elastic (Young's) modulus (*E*) and geometric moment of inertia (*I*) when modeled as a homogenous isotropic material by *κ* = *EI*. It is related to the bending persistence length (*L*_*B*_) according to *κ* = *k_B_TL*_*B*_, where *k*_*B*_ is Boltzmann's constant and *T* is the absolute temperature in Kelvin, making *L*_*B*_ a convenient proxy for filament bending stiffness. Filaments with lengths much shorter than *L*_*B*_ behave as rigid rods, as flexible polymers when they are much longer *L*_*B*_, and as semiflexible polymers at lengths comparable with *L*_*B*_.

To determine the bending stiffness of TgAct1 filaments, we visualized filaments that had been directly adsorbed to a functionalized glass coverslip. The resulting filament orientations represent a “snapshot” of the thermally driven filament shape distribution in solution (24, 25). This static measurement should yield the equivalent results as directly measuring 2D filament shape fluctuations in real time (26, 27).

Adapting this method to TgAct1 filaments requires several considerations due to its unique properties (1): TgAct1 remains filamentous for only ∼1 h after initiating polymerization before ATP is depleted and filaments depolymerize (2), TgAct1 filaments rapidly depolymerize when diluted below their critical concentration, and (3) the only known fluorescent reporter that binds TgAct1 is an actin chromobody fused to a fluorescent protein (CB-EmFP). Conventional methods to label TgAct1 such as copolymerization with fluorescently labeled monomers cannot be used to visualize TgAct1 filaments because directly labeled TgAct1 monomers do not polymerize efficiently (13). Because filaments were completely immobilized before adding the chromobody, there should be no changes in filament shape fluctuations and shape due to the increased mass (*i*.*e*., geometric moment, *I*).

**Figure 1:**
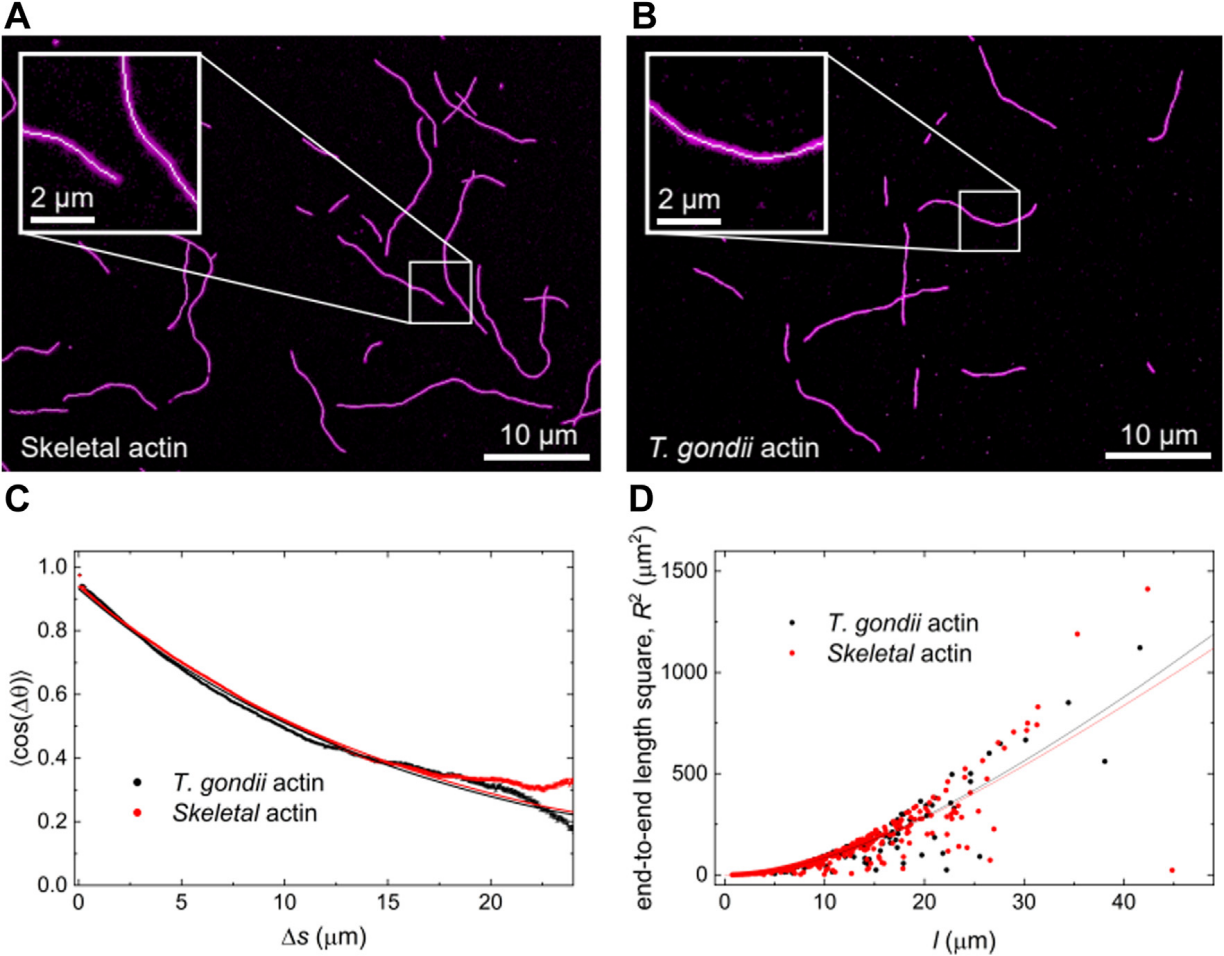
**The bending persistence lengths of *Toxoplasma gondii* (TgAct1) and vertebrate skeletal muscle actin filaments are similar.** Epifluorescence images of (*A*) skeletal muscle actin (*magenta*) and (*B*) *T*. *gondii* (TgAct1) actin (*magenta*) filaments overlayed with skeletonized traces (*white lines*). Insets are 3x magnification. Epifluorescence images were processed to facilitate skeletonization (28). *C*-*D*, the bending persistence length (*L*_B_) was determined from (*C*) a cosine correlation (the smooth, continuous line through the data (*filled circles*) represents the best fit of the data to Equation 1) or (*D*) an end-to-end distance analysis (the smooth, continuous line through the data (*filled circles*) represents the best fit of the data to Equation 2). Both analysis methods yielded comparable results (Table 1). The *L*_*B*_ value determined by cosine correlation (*C*) is 8.3 ± 0.05 for TgAct1 and 8.5 ± 0.03 μm for vertebrate skeletal muscle actin. A *p* value of 0.0006 (using the two-sample, two-tailed Z-test) for the two samples shows the *L*_*B*_ values are statistically different, though the difference is small. The *L*_*B*_ value determined from an end-to-end length analysis (*D*) is 9.3 ± 0.4 for TgAct1 and 8.5 ± 0. 3 μm for vertebrate skeletal muscle actin. A *p* value of 0.11 (using the two-sample, two-tailed Z-test) for the two samples confirms the *L*_*B*_ values are not statistically different. The uncertainty in *L*_*B*_ values represents the standard error from the fit. The data shown are from three separate and independent experiments, which yielded similar results. The sample size of the data used for analysis is 1059 skeletal and 397 TgAct1 actin filaments.

Due to these unique properties that render TgAct1 filaments short-lived and require secondary components for visualization, we immobilized TgAct1 filaments onto a blocked coverslip functionalized with *N*-ethylmaleimide (NEM)-treated muscle myosin before labeling with CB-EmFP and imaging by fluorescence microscopy (13). A higher concentration of NEM-treated muscle myosin was used than in our previous study (13) to completely immobilize filaments and to inhibit filament depolymerization, as shown using time-lapse imaging (Movie S1). As a control, the same immobilization procedure was performed for freshly polymerized skeletal muscle actin. This procedure resulted in the attachment and visualization of long filaments with low background for both filament types (Fig. 1, *A* and *B*).

The bending persistence length (*L*_*B*_) of TgAct1 and skeletal muscle actin filaments were determined from fluorescent filament images using two methods: cosine correlation and end-to-end distance analysis. Cosine correlation determines the persistence length by measuring the changes in the cosine of the tangent angle (Δ*θ*) between two points separated by a contour segment length (Δ*s*) along a filament (Fig. S1) according to (16, 20, 27, 28):

(1) $$\begin{array}{c} \langle \cos\left(\Delta \theta \right)\rangle =e^{-\frac{\Delta s}{2L_{B}}} \end{array}$$

From a cosine correlation analysis, the *L*_*B*_ of TgAct1 filaments (8.3 ± 0.05 μm) is nearly identical to that of vertebrate skeletal muscle actin (8.5 ± 0.03 μm), both measured here in parallel using identical methods (Fig. 1*C*, Table 1). The *L*_*B*_ values of vertebrate actin obtained here by visualization with fluorescent CB-EmFP are consistent with published values determined for skeletal muscle actin (without phalloidin) using various methods by different groups (16, 27, 28, 29, 30, 31).

**Table 1:** Filament bending persistence lengths (*L_B_*)

Actin isoform	*L_B_* (μm)	Method	Reference
TgAct1 (native)	8.3 ± 0.05	Cosine correlation	This study, Figure 1*C*
TgAct1 (native)	9.3 ± 0.4	End-to-end distance	This study, Figure 1*D*
Vertebrate (native)	8.5 ± 0.03	Cosine correlation	This study, Figure 1*C*
Vertebrate (native)	8.5 ± 0.3	End-to-end distance	This study, Figure 1*D*
Vertebrate (native)	8.8 ± 0.9	Cosine correlation	(28)
Vertebrate (native)	10.8 ± 1.8	End-to-end distance	(28)
Vertebrate (+phalloidin)	17	Transverse fluctuation	(16)
Vertebrate (+phalloidin)	17	Cosine correlation	(16)
Vertebrate (native)	9	Transverse fluctuation	(16)
Vertebrate (native)	9	Cosine correlation	(16)

Note: The uncertainties in *L*_*B*_ values determined in this work in the first four rows represent the standard errors from the best fits of the data.

We also determined the filament *L*_B_ values using an end-to-end distance analysis that measures how the mean squared distance between the two filament ends correlates with its contour length. This end-to-end analysis determines *L*_*B*_ from the global shape of the filament and is a complementary method to the cosine correlation analysis. Through this analysis, *L*_*B*_ can be determined by fitting a plot of the mean square end-to-end distance (*R*^2^) *versus* filament contour length (*l*) using:

(2) $$\begin{array}{c} R^{2}=8L_{B}^{2}\left(\frac{l}{2L_{B}}-1+e^{-\frac{l}{2L_{B}}}\right) \end{array}$$

We note that this equation used here for 2D bending differs from the 3D form (20, 28, 32). Here, the *L*_*B*_ term in the 3D form is replaced with 2*L*_*B*_ for the 2D form, similar to the cosine correlation analysis in 2D and 3D forms (see Methods for derivation).

The *L*_*B*_ of TgAct1 filaments (9.3 ± 0.4 μm) determined from end-to-end distance analysis is comparable to that of skeletal muscle actin filaments (8.5 ± 0.3 μm; Fig. 1*D*, Table 1). The similar *L*_*B*_ values obtained by cosine correlation and end-to-end distance analysis provide confidence in bending stiffness values of TgAct1 and skeletal muscle actin filaments under our experimental conditions.

### TgAct1 actin filaments have a similar length distribution and thermodynamic stability as skeletal muscle actin filaments

**Figure 2:**
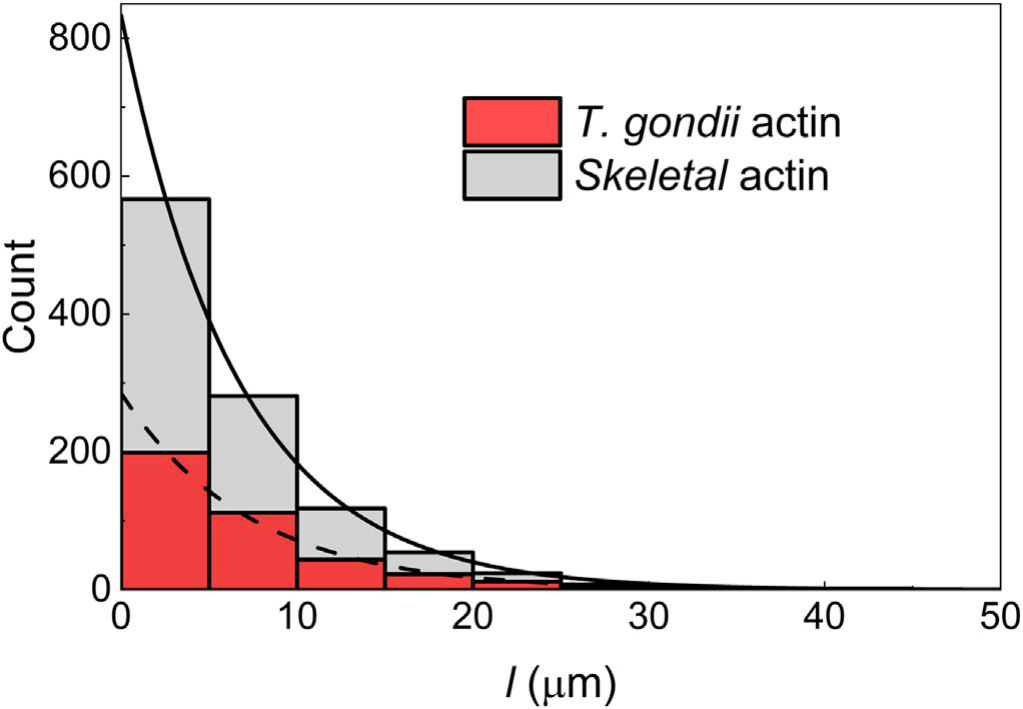
**Length distributions of *Toxoplasma gondii* (TgAct1) and skeletal muscle actin filaments.** Filament length distribution histograms of skeletal muscle (*gray*) and TgAct1 (*red*) actin show that the number of filaments of a given length decays exponentially with the length. The characteristic decay parameter (*λ*) is 0.14 ± 0.006 μm^−1^ for TgAct1 actin (*dashed line*) and 0.15 ± 0.003 μm^−1^ for skeletal muscle actin (*solid line*), determined by fitting the histogram to the exponential function, $P\left(l\right)=\frac{N}{\lambda }e^{-\lambda l}$ (Equation 6) with *N* being the total number of filaments. The uncertainty in the *λ* values represents the standard errors in the fit. A *p* value of 0.14 (using the two sample, two tailed Z-test) indicates the *λ* values are not statistically different. The average filament length ($\bar{l}$), calculated according to Equation 11, is 7.1 ± 0.3 μm for TgAct1 and 6.7 ± 01 μm for vertebrate skeletal muscle actin, respectively, and the corresponding average filament length obtained by direct arithmetic averaging over all observed filaments is 6.9 ± 0.3 and 6.5 ± 0.2 μm, respectively (Table 2). The uncertainties calculated for $\bar{l}$ from *λ*^-1^ originate from error propagation of the standard errors in *λ*, while the uncertainties in $\bar{l}$ calculated from direct arithmetic averaging represent SDs. Since the *λ* values for the two samples are not statistically different, the calculated $\bar{l}$ values calculated from *λ* are also not statistically different. However, the $\bar{l}$ values of the two actin types calculated by direct arithmetic averaging have a *p* value ∼0 (calculated using the two-sample, two-tailed Z-test), such that the two $\bar{l}$ values are statistically different. The data shown are from three separate and independent experiments with a sample size of 1059 skeletal and 397 TgAct1 actin filaments.

To gain further information about the thermodynamic stability of TgAct1 actin filament polymerization, we measured its filament length distribution (Fig. 2, red). As a control, the length distribution of skeletal muscle actin filaments was measured in parallel (Fig. 2, gray). Both measured filament length distributions follow exponential decays with comparable characteristic “decay” parameters (*λ* = 0.14 and 0.15 μm^−1^; Fig. 2, smooth lines over the histograms) determined by fitting the filament length (*l*) distributions to a theoretical exponential distribution function (Equation (8), (9). in Methods). Accordingly, the average filament lengths, given by *λ*^−1^ (Equation 11), are also comparable for both actin types (7.1 *versus* 6.7 μm). This value is consistent with the previously reported mean filament length value of 6.7 μm determined for skeletal muscle actin under comparable solution conditions (33). We note that TgAct1 filaments disassemble rapidly in solution (13). To minimize filament disassembly, we used higher concentrations of NEM-treated muscle myosin than our previous study (13), which completely immobilized filaments and prevented filament disassembly, as verified by direct visualization (Movie S1).

The average filament lengths calculated this way are very close to the values obtained from the arithmetic mean of all measured filament lengths, with differences of only ∼3 percent for both actins (Fig. 2 and Table 2). This similarity indicates that short filaments below the detection limit are not significantly influencing the measured average filament lengths; if short filaments under the detection limit were present significantly but not accounted for, the arithmetic mean of measured filament lengths would differ from the mean length.

**Table 2:** Polymerization parameters

Parameter	TgAct1	Vertebrate	Reference
*λ* (μm^−1^)	0.14 ± 0.006	0.15 ± 0.003	Figure 2
$\bar{l}$ (μm) by $\frac{1}{\lambda }$	7.1 ± 0.3	6.7 ± 01	Figure 2
$\bar{l}$ (μm) as arithmetic average	6.9 ± 0.3	6.5 ± 0.2	Figure 2
$\bar{l}$ (μm)		6.7	(33)
*K*_*pol*_ (μM; Equation 4)∗	10.3 ± 1.6		*k*_+_ and *k*_*−*_ are from (13)
*K*_*pol*_ (μM; Equation 4)∗		0.16 ± 0.10	*k*_+_ and *k*_*−*_ are from (14)
*K*_*pol*_ (μM; Equation 4)∗		0.17	*k*_+_ and *k*_*−*_ are from (15)
$a_{c}$ (μM)	10.3		As calculated in footnote
$a_{c}$ (μM)		0.13 ± 0.08	Pyrene actin (55)

*Note* ∗: $a_{c}$ = 0.9996 *K*_*pol*_ ∼ *K*_*pol*_ (Equation 6). The uncertainty in *λ* is the standard error from the fit to Equation 6; the uncertainty in $\bar{l}$ calculated from the inverse of *λ* is propagated error from *λ*, and that in from direct arithmetic average is SD.

The free energy change associated with subunit incorporation determines the filament length distribution and average length at steady state (34) and chapter 3 in (35), where the term *κ* in Equation 1 of (34) is indicated by *λ*Δ*l* here in Equation (8), (9). Accordingly, the similar observed filament length distribution and average filament lengths indicate that TgAct1 and skeletal muscle actin filaments have a similar free energy change for each incorporated subunit and therefore similar thermodynamic stabilities. For comparison, if the filament length distribution (Fig. 2) were to decay 10 times faster with a *λ* value of 1.4 μm^−1^, the average filament length would be 10 times shorter (1/1.4 ∼ 0.7 μm) because the free energy for subunit incorporation is 10 times higher such that the filaments become much unstable.

### TgAct1 actin filaments have a >60-fold weaker polymerization constant (K_pol_) than skeletal muscle actin filaments

The critical concentration is the concentration of free monomers in equilibrium with the polymer. When both barbed and pointed filament ends are available and monomers bind the two ends with different affinities (*k*-/*k*_+_), the total free monomer concentration in solution in equilibrium with filaments is a value intermediate between these two affinities. At steady state, the overall polymerization equilibrium constant for monomer incorporation at both filament ends (*K*_*pol*_) is given by the sum of contributions from filament barbed- and pointed-ends according to (36):

(3) $$\begin{array}{c} K_{pol}=\frac{k_{-}^{overall}}{k_{+}^{overall}}=\frac{k_{-}^{barbed}+k_{-}^{pointed}}{k_{+}^{barbed}+k_{+}^{pointed}} \end{array}$$

where *k*_+_ and *k*_*−*_ are the association and dissociation rate constants for subunit incorporation at filament ends.

Given the previously measured average elongation rate constants for TgAct1 polymerization at barbed and pointed ends in the presence of ATP (13), TgAct1 filaments have a *K*_*pol*_ value of 10.3 ± 1.6 μM (Equation (8), (9); Table 2). The elongation rate constants of vertebrate skeletal muscle actin yield a *K*_*pol*_ value of 0.16 ± 0.10 μM (14) and 0.17 μM (15) (Table 2). Therefore, TgAct1 has a >60-fold larger *K*_*pol*_ than skeletal muscle actin, and smaller standard free energy change (Δ*G*^o′^) associated with incoming monomer binding to both filament ends, even though both have similar mechanical properties and thermodynamic stabilities. In other words, the subunit energies and mechanics *within* filaments are identical for TgAct1 and skeletal muscle actin filaments, but the filament end binding affinities differ dramatically.

## Discussion

### Contributions of the TgAct1 actin D-loop to filament bending stiffness

Structural analysis of vertebrate actin has revealed that the D-loop of actin subdomain 2 makes stabilizing interactions with neighboring subunits along the same protofilament (longitudinal, long-pitch, intraprotofilament interaction (19, 37)) and the adjacent protofilament (lateral, short-pitch interprotofilament interaction (37)). The D-loop is highly dynamic, adopting multiple conformations, including bound, partially bound, and “open” or unbound (24, 37, 38). Molecular dynamics simulations indicate that the D-loop's conformation could also modulate skeletal muscle actin filament mechanical properties (39, 40, 41). Its ability to make longitudinal and lateral stabilizing interactions with the adjacent intraprotofilament and interprotofilament subunits suggests that the D-loop conformation could potentially affect filament assembly properties (37).

The actin D-loop sequence of TgAct1 (residues 40-KNPGIMVGMEEK-51) differs from that of skeletal muscle actin (residues 39-RHQGVMVGMGQK-50) by six amino acids. These substitutions compromise backbone and hydrogen bonding contacts between the TgAct1 D-loop and its binding pocket in the longitudinally adjacent filament subunit (13). Given that stabilizing intersubunit interactions are mediated by the D-loop, one might expect these changes to alter the filament mechanical properties.

We found minimal differences in bending mechanical properties between TgAct1 and skeletal muscle actin filaments (Fig. 1 and Table 1), despite the considerable changes in D-loop composition and contacts. A potential explanation is that D-loop interactions only modestly contribute to overall actin filament bending mechanics and the bending stiffness is largely determined and dominated by the many other longitudinal and lateral contacts made between neighboring filament subunits. This is consistent with mesoscopic-length scale models of actin filaments (42, 43, 44, 45) that capture experimentally observed filament mechanical properties and show contacts at the outer radius of filaments rupture most easily, particularly under load (45, 46). Alternatively, the compromised D-loop interactions in TgAct1 filaments may be compensated by other stabilizing interactions (discussed below).

### Intersubunit cation-binding sites and salt bridges and are conserved in TgAct1 filaments

The mechanical stability of skeletal muscle actin is determined in part by “stiffness cation” binding at a site-specific site positioned between adjacent filament subunits (30). Cation occupancy at this site is linked to formation of a salt bridge between E167 of one subunit and K61 in the neighboring subunit (29, 47), such that cation binding between Y166 and the side chain oxygen of S60 of adjacent subunits (19) promotes the allosteric formation of this salt bridge between adjacent actin filament subunits (19). Filaments with E167 and K61 do not stiffen with cations (47), consistent with the salt bridge not forming in low salt buffer when the “stiffness cation” binding site is vacant (19).

**Figure 3:**
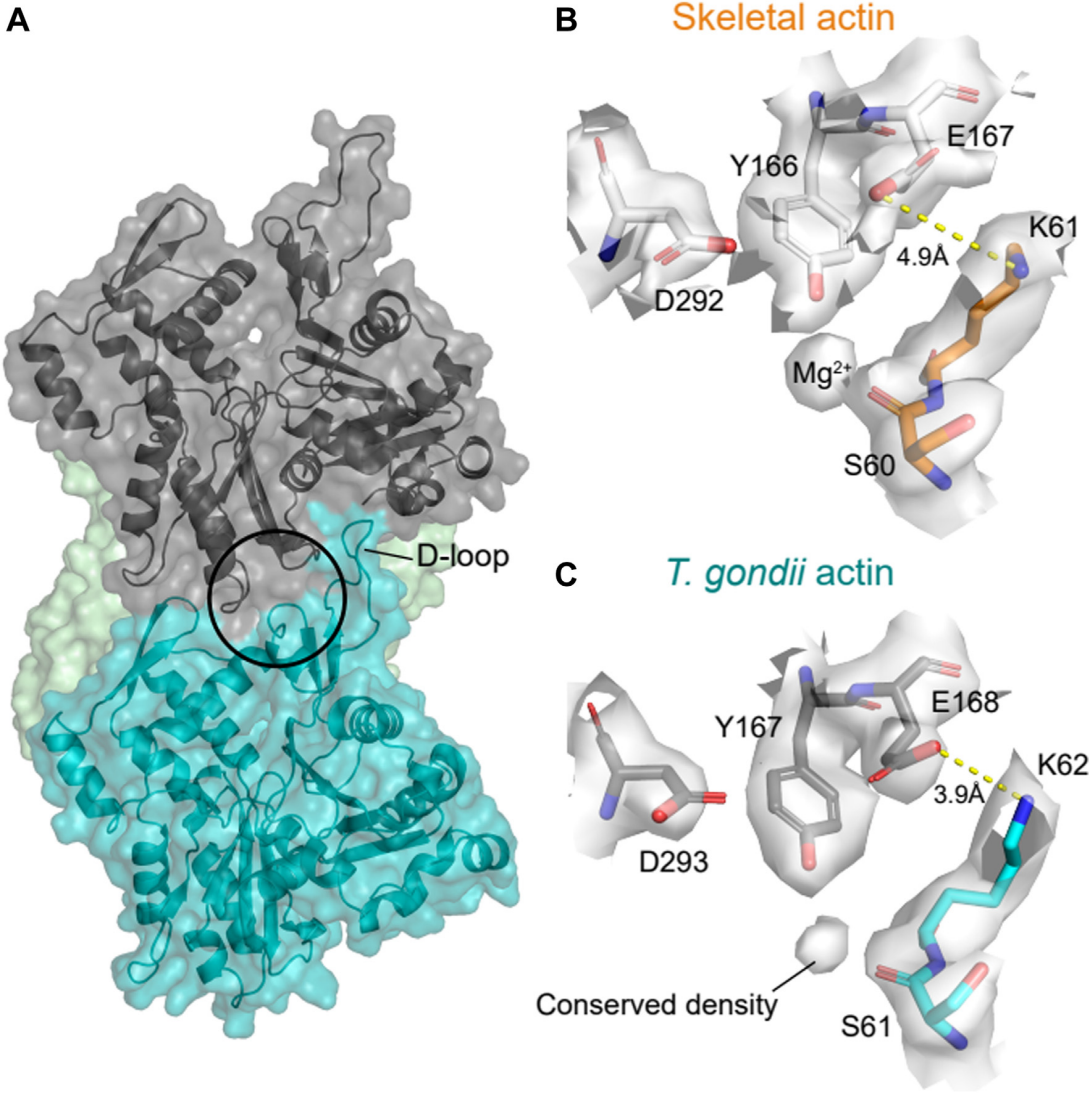
**Comparison of cation binding sites for skeletal muscle and *Toxoplasma gondii* (TgAct1) actin filaments**. *A*, surface representation of TgAct1 actin (PDB code: 8TRM) indicating the location of the “stiffness cation” site located at the filament subunit interface (*circle*). *B*-*C*, comparison of electron density surrounding the stiffness site of (*B*) skeletal muscle (PDB code: 8A2R) and (*C*) TgAct1 actin filaments. The location of the cation density coordinated by Y166 and S60 in skeletal muscle actin (19) is present in TgAct1 actin. In addition, a critical stabilizing salt bridge formed between E167 and K61 in skeletal muscle actin (*yellow dashed line*) is also formed in TgAct1 actin, with distances between residues of 4.9 Å and 3.9 Å, respectively.

Structural analysis of TgAct1 filaments reveals that the E167/K61 salt bridge in skeletal muscle actin (E168/K62 in TgAct1) is conserved (Fig. 3, *B* and *C*). Consistent with the stiffness cation-binding site also being conserved and occupied, a pocket between the residues important for cation coordination in skeletal muscle actin (Y166/S60 (Fig. 3*B*),) is observed in TgAct1 filaments (Y167/S61; Fig. 3*C*), with cryo-EM maps showing electron density in the pocket between Y167 and S61 (Fig. 3*C*) in the same position (Fig. 3*B*) as the stiffness cation found in skeletal muscle actin (19).

These conserved cation binding and salt bridge interactions are expected to contribute to the bending mechanical properties of TgAct1 actin as they do for skeletal muscle actin filaments (29, 30, 47). It is possible that the D-loop still plays important roles in TgAct1 filament mechanics, as reported for skeletal muscle actin, but the compromised D-loop contacts observed in the TgAct1 filament structure are compensated by stronger salt bridge and cation-binding site interactions. Consistent with this behavior, the E168/K62 residues forming a salt bridge in TgAct1 filaments are positioned closer together (3.9 Å) compared to their counterparts in skeletal muscle actin (4.9 Å), which may reflect a more stable salt bridge that promotes linked cation binding (Fig. 3).

### Why is the critical concentration of TgAct1 much larger than that of vertebrate muscle actin?

TgAct1 and skeletal muscle actin filaments have similar length distributions and average lengths (Fig. 2), which means subunits within both filaments have similar thermodynamic stabilities (34). The nearly identical structures of both filaments (13) support this finding. In contrast, the TgAct1 critical concentration is ∼60-fold higher than that of skeletal muscle actin (Table 2; (13)), indicating that subunits incorporating at filament ends differ considerably in their thermodynamic stability than subunits within filaments.

Terminal subunits of actin filaments have fewer lateral and longitudinal contacts and adopt unique configurations that differ from internal subunits within filaments (20, 21, 48). The D-loop of an incoming subunit tethers it to long-axis neighbor at the barbed end, suggesting the weak TgAct1 D-loop interactions visualized in the filament structure also weaken terminal subunit tethering. Consistent with this behavior, substituting the D-loop of skeletal muscle actin into *Plasmodium* actin, which displays a high critical concentration like TgAct1, lowers the critical concentration from ∼4 μM to ∼0.9 μM (49). Presumably, compromised D-loop contacts also contribute to the weaker pointed end critical concentration of TgAct1. Collectively, these results indicate that changes in the TgAct1 D-loop significantly influence interactions between incoming monomers and filament ends but not between internal subunits within filaments.

### Relationship between filament bending stiffness, the critical concentration, and internal subunit binding free energy

Although the actin filament stiffness is undoubtedly influenced by the thermodynamics of intersubunit interactions (20), the work presented here demonstrates that there is no strict relationship between the critical concentration and actin filament mechanical properties. Consistent with this being a general behavior, GMPCPP-tubulin has a 100-fold smaller critical concentration than GDP-tubulin, but only a 2x higher bending stiffness (50). Similarly, adding taxol lowers the microtubule critical concentration by about 100-fold but the bending stiffness changes modestly (50). This relationship breaks down because the argument that the internal subunit interaction energy can be inferred from the critical concentration is flawed; the internal subunit interaction free energy and the standard free energy change calculated from the critical concentration are different.

## Methods

### Preparation of actin filaments for imaging by epifluorescence microscopy

TgAct1 (untagged) was purified as described (13), dialyzed into storage buffer (5 mM Tris, pH 8.2, 0.2 M ammonium acetate, 0.25 mM CaCl_2_, 0.5 mM DTT, and 0.25 mM Na_2_ATP), and stored at −80 °C. A 50 μl aliquot of TgAct1 was thawed briefly in room temperature (RT) water and clarified at 400,000×*g* for 30 min. before exchanging into G buffer (5 mM Tris, pH 8.2, 0.25 mM CaCl_2_, 0.5 mM DTT, 0.25 mM Na_2_ATP) by passing over a Zeba spin-desalting column (Thermo Fisher Scientific 89882).

TgAct1 was immediately polymerized by adding 0.1 volumes of 10x polymerization buffer (250 mM imidazole, pH 7.4, 500 mM KCl, 20 mM MgCl_2_, 10 mM EGTA, 2 mM MgATP, and 10 mM DTT) and incubated at 37 °C for 45 min. Using a positive displacement pipette, freshly polymerized actin filaments were diluted to 100 nM in 0.5 ml RT buffer A (25 mM imidazole pH 7.4, 1 mM EGTA, 1 mM DTT, 50 mM KCl, and 2 mM MgCl_2_) and mixed gently by manual inversion four times. Then, 500 μl were pipetted onto a 22 × 22 mm coverslip that was adsorbed with 0.004 mg mL^-1^ NEM-inactivated chicken skeletal muscle myosin in buffer M (25 mM imidazole (pH 7.4), 250 mM KCl, 1 mM EGTA, and 4 mM MgCl_2_; (51)), equilibrated for 2 min, then blocked with buffer B (25 mM imidazole, pH 7.4, 50 mM KCl, 2.5 mM MgCl_2_, 1 mM EGTA, and 10 mM DTT) containing 5 mg ml^-1^ bovine serum albumin and 1% Pluronic F127 (Invitrogen, P6866). The coverslip was inverted onto a slide containing double-stick tape to form a flow chamber. The chamber was washed 3x with buffer B followed by two passages of buffer B containing 100 nM actin and 50 nM CB-EmFP and imaged immediately by epifluorescence microscopy using a DeltaVision Elite microscope (Cytiva) built on an Olympus base equipped with a 100 × 1.39 NA objective, scientific CMOS camera, and DV Insight solid-state illumination module. The resulting NEM-treated myosin density was sufficient to completely immobilize actin filaments, as observed using time-lapse imaging (Movie S1).

Skeletal muscle actin was purified from chicken pectoralis acetone powder (52) and stored as monomers in liquid nitrogen. Filament samples were prepared in parallel with TgAct1 filaments using a similar method, except desalting was not required as the actin is purified in buffer A, polymerization was performed at room temperature for 1 h, and filaments were diluted to 20 nM before attachment.

### Determining the bending persistence lengths (L_B_) of TgAct1 and skeletal actin filaments

Polymerized filament samples were adsorbed onto a blocked coverslip functionalized with NEM-treated muscle myosin before labeling with 50 nM CB-EmFP and imaging by fluorescence microscopy as described (13). Fluorescent filament images were processed to remove background noise and then skeletonized to facilitate automated tracking (28).

Cosine correlation and end-to-end distance analyses were done using *Persistence* software (freely downloaded from https://delacruzlab.yale.edu/persistence-software) as described in detail (28). We note the end-to-end analysis in (28) is for 3-D fluctuations, whereas here filaments are in-plane so we analyze data using the 2D form of the relationship that we derive here parallel to the derivation for 3D form (20, 32):

The vector from one end of a polymer to the other ($\vec{R}$) is given by (32):

(4) $$\begin{array}{c} \vec{R}=\int_{0}^{l}\hat{t}ds \end{array}$$

where $\hat{t}\left(s\right)$ is the tangent vector at position (*s*) following the integration along the polymer contour from one end (*s* = 0) to the other end (*s* = polymer contour length *l*). The average of end-to-end length squared ($\langle R^{2}\rangle$) is given by the average dot product of $\vec{R}$ which is derived according to the following procedure:

$$\langle R^{2}\rangle =\langle \vec{R}\cdot \vec{R}\rangle =\int_{0}^{l}\int_{0}^{l}\langle {\hat{t}}_{1}\cdot {\hat{t}}_{2}\rangle ds_{1}ds_{2}$$

$$=\int_{0}^{l}\int_{0}^{l}\langle \cos\Delta \theta \rangle ds_{1}ds_{2}=\int_{0}^{l}\int_{0}^{l}e^{-\frac{\left|s_{1}-s_{2}\right|}{2L_{B}}}ds_{1}ds_{2}$$

$$=\int_{0}^{l}ds_{1}\left(\int_{0}^{s_{1}}e^{-\frac{s_{1}-s_{2}}{2L_{B}}}ds_{2}+\int_{s_{1}}^{l}e^{-\frac{s_{2}-s_{1}}{2L_{B}}}ds_{2}\right)$$

$$=2L_{B}\int_{0}^{l}ds_{1}\left(e^{-\frac{s_{1}}{2L_{B}}}\left(e^{\frac{s_{1}}{2L_{B}}}-1\right)-e^{\frac{s_{1}}{2L_{B}}}\left(e^{-\frac{l}{2L_{B}}}-e^{-\frac{s_{1}}{2L_{B}}}\right)\right)$$

$$=2L_{B}\int_{0}^{l}ds_{1}\left(2-e^{-\frac{s_{1}}{2L_{B}}}-e^{-\frac{l}{2L_{B}}}e^{\frac{s_{1}}{2L_{B}}}\right)$$

$$=2L_{B}\left(2l+2L_{B}\left(e^{-\frac{l}{2L_{B}}}-1\right)-2L_{B}e^{-\frac{l}{2L_{B}}}\left(e^{\frac{l}{2L_{B}}}-1\right)\right)$$

(5) $$\begin{array}{c} =2L_{B}\left(2l-4L_{B}+{4L_{B}e}^{-\frac{l}{2L_{B}}}\right)=8L_{B}^{2}\left(\frac{l}{2L_{B}}-1+e^{-\frac{l}{2L_{B}}}\right)\; \end{array}$$

where

$${\hat{t}}_{1}\cdot {\hat{t}}_{2}=\cos\Delta \theta =\cos\left(\theta_{2}-\theta_{1}\right)$$

and the 2D cosine correlation function (Equation 1) are used. The outcome of the derivation shown in Equation (8), (9) is the same as Equation 2 used in the Results section.

### Filament length analysis

According to polymerization theory ((34, 36, 53), chapter 9 in (20), chapter 3 in (35)), the polymerized actin filament contour length distribution follows a power law distribution. That is, a power function of filament length *j* in the number of subunits according to:

(6) $$\begin{array}{c} P\left(j\right)=\text{const}.{\left(\frac{a_{c}}{K_{pol}}\right)}^{j}=\text{const}.e^{j\;\ln\left(\frac{a_{c}}{K_{pol}}\right)}=\text{const}.e^{l\;\ln\left(\frac{a_{c}}{K_{pol}}\right)/\Delta l}=\frac{N}{\lambda }e^{-\lambda l}=P\left(l\right)\; \end{array}$$

where *P*(*j*) is concentration of filaments *j* subunits long; const. is a constant introduced as a scaling factor for fitting experimental data; $a_{c}$ is the critical monomer concentration coexisting with in a reversible equilibrium with assembled filaments; and Δ*l* is the segment length of a filament subunit and is ∼ 2.7 nm for both skeletal muscle and TgAct1 filaments. The length (*l*) of a filament with *j* subunits is *l* = *j*Δ*l*. By converting *j* in subunits to *l* in μm, the distribution *P*(*j*) as function of *j* becomes an exponential distribution as function of filament contour length (*l*; Equation (8), (9)), as observed experimentally here and previously reported (33, 54). *K*_*pol*_ is the overall polymerization equilibrium constant for monomer incorporation to polymer at the both barbed and pointed ends (Equation (8), (9)) in Results section and we also presented here (Equation 7 below) for convenience since it is an important quantity in Equation (8), (9). and Equations (8), (9) below.

(7) $$\begin{array}{c} K_{pol}=\frac{k_{-}}{k_{+}}=\frac{k_{-}^{barbed}+k_{-}^{pointed}}{k_{+}^{barbed}+k_{+}^{pointed}} \end{array}$$

The exponential distribution decay parameter (λ) in Equation (8), (9) is expressed as:

(8) $$\begin{array}{c} \lambda =-\frac{\ln\left(\frac{a_{c}}{K_{pol}}\right)}{\Delta l} \end{array}$$

which can be rewritten as:

(9) $$\begin{array}{c} \frac{a_{c}}{K_{pol}}=e^{-\lambda \Delta l} \end{array}$$

thus, indicating that the filament length scales with the ratio of the free monomer concentration ($a_{c}$) and the overall polymerization equilibrium constant for monomer incorporation at both filament ends (*K*_*pol*_). Substituting the values of *λ* and Δ*l* (Table 1) into Equation 9 yields:

$$\frac{a_{c}}{K_{pol}}=e^{-\lambda \Delta l}=0.9996∼1$$

that is,

(10) $$\begin{array}{c} a_{c}∼K_{pol} \end{array}$$

The two quantities are almost equal and differ only slightly (<0.04%) for both actins being studied here. In fact, the values of *a*_*c*_ and *K*_*pol*_ cannot be equal. If they were equal, filaments would be a constant length (*i*.*e*., *λ* = 0) and not display a distribution, as given by Equations (8), (9). The length distribution originates from this small difference between *K*_*pol*_ and $a_{c}$.

The average filament length can be calculated from the filament length distribution, Equation (8), (9):

(11) $$\begin{array}{c} \bar{l}=\frac{\int_{0}^{\infty }lP\left(l\right)dl}{\int_{0}^{\infty }P\left(l\right)dl}=\frac{\int_{0}^{\infty }le^{-\lambda l}dl}{\int_{0}^{\infty }e^{-\lambda l}dl}=\frac{1}{\lambda } \end{array}$$

## Data availability

All original data underlying the findings described in this article are fully available upon request to the corresponding author (Enrique M. De La Cruz; enrique.delacruz@yale.edu).

## Supporting information

This article contains supporting information.

## Conflict of interest

The authors declare that they have no conflicts of interest with the contents of this article.
