# Supplementary material for: Bending stiffness of Toxoplasma gondii actin filaments
Source: J Biol Chem. 2024 Dec 18;301(2):108101. doi: 10.1016/j.jbc.2024.108101 (PMC11786770; doi:10.1016/j.jbc.2024.108101)
Supplement: Supplementary Figure S1 [file mmc1.pdf]

Supplementary Information

Bending stiffness of *Toxoplasma gondii* actin filaments

Wenxiang Cao[1]*, Thomas E. Sladewski[2]*, Aoife T. Heaslip[2], Enrique M. De La Cruz[1†]

*Equal contributing authors

1. Department of Molecular Biophysics and Biochemistry, Yale University, New Haven, CT 06520.
2. Department of Molecular and Cell Biology, University of Connecticut, Storrs, Connecticut, USA

†Corresponding author, e-mail: enrique.delacruz@yale.edu

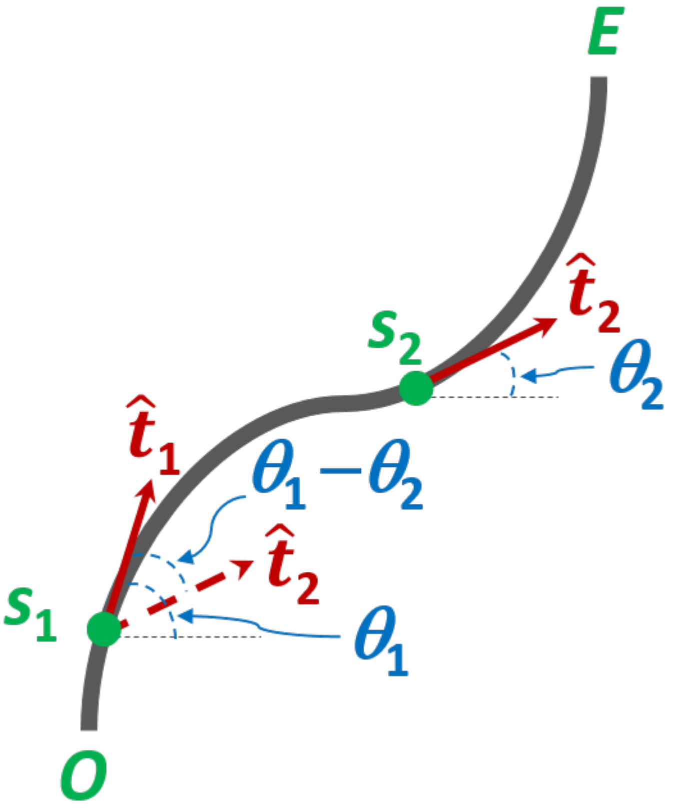

Supplementary Figure S1: Illustration of unit tangent vectors, tangent angles, and segment lengths along a filament used for bending stiffness analysis with Eqs. (1) and (2). The contour length of a filament is defined as the length along the filament, starting at one end, defined as the origin *O*, and terminating at the other end, defined by point *E*. There are two points indicated along the filament at positions s_1 and s_2. The contour distance is defined as the distance along the filament from the origin *O*. The distance between the two points along the contour is $\Delta s = s_1 - s_2$. The unit vectors along the tangent lines (unit tangent vectors) at the two points are $\hat{t}_1$ and $\hat{t}_2$, and their tangent angles with respect to the horizontal line are given by θ_1 and θ_2, respectively. The dashed unit tangent vector $\hat{t}_2$is the same as vector $\hat{t}_2$, but is moved from position s_2 to s_1 to illustrate the tangent angle difference ($\Delta\theta$) between the two tangent vectors at the two different positions, defined by $\Delta\theta = \theta_1 - \theta_2$. The variables Δs and $\Delta\theta$ are used in Eq. (1) for calculating the cosine correlation between the two points. The linear distance between the filament origin (*O*) and the other end (*E*) defines the end-to-end length (*R*), which is used with the filament contour length between *O* and *E* (*l*) in Eq. (2).

Supplementary Movie S1: Time lapse movie showing TgAct1 filaments immobilized on the surface by NEM-treated muscle myosin. Conditions: 25 mM imidazole (pH 7.4), 50 mM KCl, 2 mM $MgCl_2$, 1 mM EGTA, 10 mM DTT, 37 °C. 10x playback, Image width: 54 µm, Image interval: 1 sec, Image duration: 30 sec.
